# Alpha-lipoic acid as a trigger of insulin autoimmune syndrome: Current evidence, a case-based approach, diagnostic pitfalls, and practical management

**DOI:** 10.1016/j.metop.2026.100491

**Published:** 2026-08-14

**Authors:** Natalia G. Vallianou, Dimitris C. Kounatidis, Maria Dalamaga, Fotis Panagopoulos, Maria Psaroudaki, Ioanna Tantsi, Theodora Stratigou

**Affiliations:** a First Department of Internal Medicine, Sismanogleio General Hospital, Athens, 15126, Greece; b Department of Pharmacology, Medical School, National and Kapodistrian University of Athens, Athens, 11527, Greece; c Department of Biological Chemistry, Medical School, National and Kapodistrian University of Athens, Athens, 11527, Greece; d Diabetes and Endocrinology Department, Evangelismos General Hospital, Athens, 10676, Greece

**Keywords:** Alpha-lipoic acid, Insulin autoimmune syndrome, Drug-induced insulin autoimmune syndrome, Glucocorticoids, Hirata disease, Hyperinsulinemic hypoglycemia, Insulin autoantibodies

## Abstract

Insulin autoimmune syndrome (IAS), also known as Hirata disease, is a rare immune-mediated cause of endogenous hyperinsulinemic hypoglycemia characterized by circulating insulin autoantibodies (IAAs). It typically develops in individuals without prior exposure to exogenous insulin. Although IAS has been associated with viral infections and, less commonly, with autoimmune and hematologic disorders, medications and dietary supplements are considered the most frequent precipitating factors. Sulfhydryl-containing compounds are the most commonly implicated triggers. Methimazole represents the classical drug-associated cause, whereas an increasing number of cases have been attributed to the widely available antioxidant supplement alpha-lipoic acid (ALA).In genetically susceptible individuals, i.e. in those carrying the HLA alleles HLA-DRB1*04:06/04:/03/04:04, sulfhydryl-containing compounds are thought to alter insulin immunogenicity and promote the formation of IAAs. The resulting insulin–antibody complexes disrupt normal insulin kinetics, leading to recurrent hypoglycemia that may alternate with episodes of postprandial hyperglycemia. Early recognition of the syndrome is of paramount importance, as withdrawal of the triggering agent together with appropriate supportive and pharmacological management is associated with a favorable prognosis in most patients. The importance of medical nutrition therapy with frequent, small meals rich in cornstarch together with the addition of acarbose or glucocorticoids should be pointed out. This narrative review provides an overview of the current evidence regarding IAS, with particular emphasis on drug- and ALA-associated disease. We further illustrate the practical application of this evidence through the presentation of a 76-year-old man who developed IAS following ALA supplementation. To our knowledge, this is the first narrative review to comprehensively address the epidemiology, pathogenesis, diagnosis, and management of ALA-associated IAS while integrating a representative clinical case to bridge current evidence with practical clinical decision-making. By highlighting this rare but increasingly recognized cause of hypoglycemia, this work aims to enhance clinical awareness and support timely diagnosis and evidence-based management.

## 1 Introduction

Insulin autoimmune syndrome (IAS), also known as Hirata disease, is a rare immune-mediated disorder first described by Hirata et al., in 1970 and characterized by spontaneous hyperinsulinemic hypoglycemia. Hypoglycemia results from the formation of autoantibodies directed against endogenous insulin and typically occurs in individuals with no prior exposure to exogenous insulin. However, cases arising after exposure to exogenous insulin have also been reported, particularly among individuals with type 2 diabetes (T2D) [1,2]. Although considered uncommon outside East Asia, increasing awareness of the syndrome and broader availability of autoantibody testing have led to a growing number of reported cases worldwide [1,3].

IAS has been associated with a variety of conditions, including viral infections, autoimmune diseases, and hematologic disorders. However, medications are considered the most common precipitating factors, with methimazole representing the most frequently implicated agent [4]. Although numerous drugs have been linked to the development of IAS, a growing number of cases have also been associated with alpha-lipoic acid (ALA), an antioxidant compound widely used both as a dietary supplement and in patients with diabetic peripheral neuropathy (DPN) [5,6]. In many of these cases, sulfhydryl-containing compounds are believed to promote the formation of insulin autoantibodies (IAAs) through the disruption of insulin disulfide bonds, enhancing immunogenicity in genetically susceptible individuals [1,3].

These autoantibodies are typically characterized by high binding capacity and relatively low affinity for insulin, facilitating the formation of circulating insulin–IAA immune complexes. This process allows large amounts of insulin to be sequestered after secretion and subsequently released in an unpredictable manner, ultimately resulting in postprandial hypoglycemia [1,2,4]. During hypoglycemic episodes, patients typically exhibit elevated or inappropriately normal C-peptide levels, with a characteristically disproportionate insulin-to-C-peptide ratio, while serum insulin measurement usually reveals markedly elevated concentrations, often exceeding 1000 μIU/mL in severe cases [3,4,7]. Demonstration of elevated IAA titers is the cornerstone of diagnosis, whereas the differential diagnosis involves other causes of endogenous hyperinsulinemic hypoglycemia, primarily insulinoma [3,4,8].

Management principles are broadly similar across all forms of IAS and include individualized dietary modification emphasizing small, frequent, low-glycemic-index meals together with targeted interventions aimed at preventing and treating hypoglycemia. In patients with persistent disease, immunosuppressive therapy, most commonly with glucocorticoids (GCs), and in refractory cases, rituximab, may be required. Furthermore, therapeutic plasma exchange may have a role in selected severe cases. In drug- or supplement-induced IAS, withdrawal of the offending agent represents an essential component of management [4,[9], [10], [11]].

To date, no comprehensive review has specifically focused on ALA-associated IAS, despite its growing clinical relevance as ALA supplementation becomes increasingly widespread worldwide. In this review, we summarize the current evidence regarding the epidemiology, pathogenesis, diagnosis, and management of IAS, with particular emphasis on drug- and supplement-induced disease and, specifically, ALA-associated IAS. We additionally present the case of a 76-year-old man who developed IAS following ALA supplementation. By integrating contemporary evidence with a representative clinical case, we aim to provide a practical, evidence-based framework for the recognition and management of this rare but increasingly relevant cause of hypoglycemia.

## 2 Insulin autoimmune syndrome

### 2.1 Epidemiology and triggers

IAS is considered the third most common cause of spontaneous hypoglycemia in Japan, after insulinoma and extra-pancreatic tumors [12]. The true incidence of IAS in the general population remains uncertain, as available epidemiological data are limited and originate predominantly from East Asia, particularly Japan [1,3,4,8]. One of the largest epidemiological investigations was a nationwide questionnaire-based survey conducted in Japan during 2017–2018. In this study, IAS accounted for 4.9% of all cases of endogenous hyperinsulinemic hypoglycemia (22 of 447 patients) treated for hypoglycemia or hypoglycemia-related complications. The same study estimated the annual incidence of IAS in the general population at approximately 0.017 cases per 100,000 inhabitants [13].

Although IAS is generally considered more prevalent among East Asian populations than among Caucasian populations, the magnitude of this ethnic difference remains uncertain. Indeed, the number of reported cases in Caucasian individuals has increased substantially in recent years, while the apparent ethnic disparity may, at least in part, reflect underrecognition of the syndrome outside Asia, resulting in underdiagnosis and underreporting [2,4]. Available epidemiological evidence further suggests that IAS affects men and women with approximately equal frequency, with no significant sex-related differences in either age at onset or duration of hypoglycemic episodes. The syndrome predominantly affects adults older than 40 years of age and is only rarely encountered during childhood [1,4,[14], [15], [16]].

Genetic susceptibility plays a central role in the development of IAS, with strong associations reported between the syndrome and specific Human Leukocyte Antigen (HLA) alleles. Notably, these associations appear to vary across ethnic groups. HLA-DRB1*04:06 has been identified as the predominant susceptibility allele in East Asian populations, whereas HLA-DRB1*04:03 appears to be the most frequently associated allele among Caucasian populations, with HLA-DRB1*04:04 also reported at increased frequency in non-East Asian patients [9,17].

In genetically predisposed individuals, a variety of environmental triggers may promote the development of IAS. Viral infections have been implicated in a number of cases, with measles, hepatitis C virus infection, and severe acute respiratory syndrome coronavirus 2 (SARS-CoV-2) among the most frequently reported examples [[18], [19], [20]]. Less commonly, IAS has been described in association with a broad spectrum of autoimmune diseases, most notably autoimmune thyroid disease, particularly Graves’ disease, as well as hematologic disorders, including monoclonal gammopathy of undetermined significance (MGUS) and multiple myeloma [3,4]. Nevertheless, the available literature consistently supports medications and dietary supplements, particularly those containing sulfhydryl groups or generating thiol metabolites, as the most common precipitating factors. In particular, compounds containing sulfhydryl (thiol) groups or thiol-derived metabolites have been most strongly associated with IAS, with methimazole, clopidogrel, and ALA representing three of the best-characterized triggers [1,3,4,21]. It should also be acknowledged that, despite extensive evaluation, no identifiable trigger can be established in a proportion of patients, a presentation sometimes referred to as idiopathic or primary IAS [4,22].

### 2.2 Clinical manifestations

The hallmark clinical manifestation of IAS is recurrent spontaneous hypoglycemia, typically presenting with a combination of autonomic and neuroglycopenic symptoms [4]. Autonomic symptoms include tremor, palpitations, sweating, anxiety, and hunger, while neuroglycopenic manifestations range from cognitive impairment and behavioral changes to seizures and coma [10]. The glycemic threshold at which symptoms occur varies considerably among affected individuals and is often lower than that observed in other causes of hypoglycemia. Likewise, the frequency, severity, and timing of hypoglycemic episodes are highly heterogeneous [4,10].

In most patients, hypoglycemia occurs in the postprandial state, typically developing 2–6 h after meal ingestion. Nevertheless, fasting and nocturnal hypoglycemia have also been reported, and a substantial proportion of patients experience both postprandial and fasting episodes [1,3,4,10]. According to available case series, approximately 42% of patients present with postprandial delayed hypoglycemia, 31% with fasting hypoglycemia, and 24% with both. Exercise-induced hypoglycemia, although uncommon, has also been described. In addition, some patients with recurrent hypoglycemia and a prolonged disease course may develop weight gain as a consequence of frequent compensatory eating [10,17].

A distinctive feature of IAS is the marked glycemic variability resulting from the kinetic properties of insulin–IAA immune complexes. Consequently, some patients experience alternating episodes of hyperglycemia and hypoglycemia. In these individuals, the characteristic pattern consists of early postprandial hyperglycemia caused by transient sequestration of insulin within circulating immune complexes, followed by delayed reactive hypoglycemia resulting from the subsequent dissociation of insulin from these complexes and the release of free biologically active insulin [4,23]. Continuous glucose monitoring (CGM) data have confirmed this alternating pattern of postprandial hyperglycemia followed by hypoglycemia and may serve as a useful adjunctive tool for characterizing glycemic variability in affected patients. Rarely, IAS may initially manifest as diabetic ketoacidosis before the onset of recurrent hypoglycemic episodes [10].

### 2.3 Diagnostic approach

The diagnostic evaluation of patients with suspected IAS begins with confirmation of hypoglycemia. As with all causes of hypoglycemia, diagnosis is traditionally based on Whipple's triad, which consists of: (i) symptoms and/or signs consistent with hypoglycemia, (ii) documented low plasma glucose concentrations, and (iii) resolution of symptoms following correction of hypoglycemia with glucose administration [24].

Once hypoglycemia has been confirmed, serum insulin concentrations should be measured during a spontaneous hypoglycemic episode. Hyperinsulinemia in IAS is often striking, with insulin concentrations frequently exceeding 100 μIU/mL and often reaching levels of 1000 μIU/mL or higher [10]. However, the degree of hyperinsulinemia varies considerably, and cases with less pronounced elevations have also been reported [3,4,25,26]. It should be noted that measured insulin concentrations in IAS may be artifactually elevated due to the interference of circulating autoantibodies with conventional immunoassays and therefore may not accurately reflect biologically active (free) insulin levels [10].

Following confirmation of hyperinsulinemic hypoglycemia, the origin of excess insulin should be determined. Measurement of C-peptide and proinsulin concentrations is useful for distinguishing endogenous from exogenous hyperinsulinism. Because IAS is, by definition, a form of endogenous hyperinsulinemic hypoglycemia, both C-peptide and proinsulin levels are typically elevated or inappropriately normal during hypoglycemia [25,26]. However, C-peptide immunoreactivity may also be artifactually increased in IAS due to the cross-reactivity of antibody-bound proinsulin (rather than true C-peptide) in some C-peptide immunoassays, a finding that has been confirmed by mass spectrometry studies [27].

The insulin-to-C-peptide molar ratio has been proposed as an ancillary diagnostic marker of IAS. Under physiological conditions, pancreatic β-cells secrete insulin and C-peptide in equimolar amounts; however, owing to their different half-lives (approximately 5–10 min for insulin and 30–35 min for C-peptide), the physiological insulin-to-C-peptide molar ratio is generally below 1 [28]. In IAS, this ratio may become elevated because of delayed insulin clearance secondary to circulating insulin–autoantibody complexes. Nevertheless, the diagnostic utility of this parameter remains controversial. No standardized analytical methods currently exist for insulin and C-peptide measurement, and results may vary according to the assay platform used [29]. Furthermore, a study of 16 patients with confirmed IAS demonstrated that only 9 had an insulin-to-C-peptide molar ratio greater than 1, while 6 had a ratio below 1, suggesting low sensitivity of this marker [30]. In addition, elevated ratios may also occur in exogenous hyperinsulinism, limiting its value in the differential diagnosis [3,29]. In their combined cohort analysis, Patel et al. demonstrated that an insulin-to-C-peptide molar ratio greater than 0.25, when measured during a hypoglycemic episode, exhibited 100% specificity and 89% sensitivity for distinguishing IAH from insulinoma, although this threshold requires further validation [9].

Detection of IAAs represents the cornerstone of diagnosis and is essential for establishing IAS. Importantly, the presence of IAAs is not invariably associated with clinical disease. Low-titer IAAs have been detected in up to 6% of healthy individuals, although this figure refers primarily to insulin autoantibodies in the context of type 1 diabetes (T1D) autoimmunity screening and may not be directly comparable to the IAAs characteristic of IAS, which differ in binding affinity and capacity [31,32]. IAAs comprise several immunoglobulin (Ig) classes, including IgG, IgM, and IgA, as well as, rarely, IgD and IgE, with IgG representing the predominant subtype. In Japan, the majority of IAS patients have polyclonal IgG IAAs, whereas monoclonal IAAs have been reported in more than half of non-Asian cases [33]. Interpretation of IAA testing requires particular caution, as many commercially available assays detect only IgG-class antibodies and may therefore yield false-negative results in patients who predominantly produce IgM- or IgA-class IAAs [34]. Consequently, a negative IAA result should not exclude the diagnosis in patients with a high clinical suspicion of IAS. In such cases, additional investigations, including polyethylene glycol (PEG) precipitation and/or gel filtration chromatography, may be required, as these methods can detect insulin-binding antibodies irrespective of Ig class [3,7].

PEG precipitation is an inexpensive and widely accessible screening method; a significant difference between total serum insulin and free insulin (measured in the supernatant after PEG precipitation) strongly supports the diagnosis of IAS. Gel filtration chromatography, while more complex and costly, provides definitive confirmation by demonstrating macromolecular insulin–antibody complexes eluting before free insulin [35,36]. Finally, mass spectrometry has also emerged as a more robust method for quantifying insulin in the presence of autoantibodies, as it is less susceptible to antibody-mediated assay interference than conventional immunoassays [36].

### 2.4 Differential diagnosis

The principal condition requiring differentiation from IAS is insulinoma, a rare functional pancreatic neuroendocrine tumor characterized by autonomous insulin secretion. Unlike IAS, hypoglycemia in patients with insulinoma occurs predominantly during fasting rather than in the postprandial state, although up to 27% of patients may present with mixed fasting and postprandial hypoglycemia, and approximately 3–6% with exclusively postprandial symptoms [37,38].

Several biochemical features may assist in distinguishing the two conditions. Serum insulin concentrations in insulinoma are generally lower than 1000 μIU/mL, whereas markedly elevated values exceeding this threshold are frequently observed in IAS [10]. Notably, a serum insulin concentration >100 μIU/mL has been reported to provide 97.5% specificity and 94% sensitivity for differentiating IAS from insulinoma [9]. Nevertheless, considerable overlap exists, and insulin concentrations should not be interpreted in isolation. In addition, IAS may be characterized by glycemic variability, with alternating episodes of hyperglycemia and hypoglycemia, while hyperglycemia is generally not observed in insulinoma [4,10].

The 72-h supervised fasting test remains the gold standard for the diagnosis of insulinoma, with approximately 98% of patients developing symptomatic hypoglycemia within 48–72 h of fasting [39]. In contrast, patients with IAS generally do not develop hypoglycemia during prolonged fasting, as hypoglycemia in IAS is typically triggered by postprandial insulin–antibody complex dynamics. However, fasting hypoglycemia has been reported in a subset of IAS patients, limiting the ability of the test to definitively exclude the diagnosis [10].

Additional clinical and laboratory features may assist in distinguishing the two conditions. Patients with insulinoma typically lack a history of exposure to medications or dietary supplements associated with IAS. In addition, IAAs are generally absent in insulinoma, although rare cases of insulinoma accompanied by detectable IAAs have been described [1,3,4,40]. The insulin-to-C-peptide molar ratio may provide supplementary information. In insulinoma, insulin and C-peptide concentrations rise in parallel, and the ratio typically remains below 1, whereas in IAS the ratio is often elevated (>1) because antibody-mediated insulin binding delays insulin clearance. However, as discussed previously in section 2.3., this parameter lacks sufficient sensitivity and specificity to serve as a standalone diagnostic marker. PEG precipitation provides another useful discriminator where insulin recovery after PEG precipitation is low (5–10%) in IAS due to antibody-bound insulin, compared with normal recovery (>70%) in insulinoma [10].

Imaging studies provide another important point of differentiation. In insulinoma, cross-sectional imaging with abdominal computed tomography (CT) or magnetic resonance imaging (MRI) frequently identifies a pancreatic lesion with sensitivities of approximately 80% for contrast-enhanced CT [41]. Functional imaging with 68Ga-DOTATATE or 68Ga-DOTATOC PET/CT may be useful in selected cases, although sensitivity for insulinoma localization is variable (26–87%), and notably two-thirds of insulinomas are Somatostatin Receptor (SSTR)-PET negative [42,43]. When available, 68Ga-exendin-4 PET/CT targeting glucagon-like peptide-1 (GLP-1) receptors has demonstrated superior sensitivity (approximately 94%) for insulinoma localization [44]. By contrast, imaging studies are typically negative in IAS and are generally not required for diagnosis unless alternative causes of endogenous hyperinsulinemic hypoglycemia cannot be confidently excluded. However, it should be noted that a small number of IAS patients may have coincidental pancreatic lesions, which may complicate the diagnostic workup [45].

Lastly, IAS is primarily managed through withdrawal of the triggering factor, individualized nutritional intervention, and, when necessary, pharmacological or immunomodulatory therapy. In contrast, surgical resection remains the definitive treatment for insulinoma [1,3,4,37]. Given the substantial differences in both pathophysiology and management, accurate distinction between these two entities is of paramount clinical importance. The major clinical, biochemical, and radiological features that help differentiate IAS from insulinoma are summarized in Table 1.

**Table 1:** Key distinguishing features between insulin autoimmune syndrome and insulinoma.

Feature	IAS	Insulinoma
**History of drug, supplement, or viral exposure**	Present in some cases	Typically, absent
**Associated comorbidities**	✔ Autoimmune disorders ✔ Hematologic diseases	Usually, absent
**Timing of hypoglycemia**	Predominantly postprandial	Predominantly fasting
**Hypoglycemia during prolonged fasting**	Typically, absent	Typically, present
**Alternating episodes of hypoglycemia and hyperglycemia**	May be present	Typically, absent
**Serum insulin concentrations**	Often markedly elevated, sometimes extremely high	Elevated, but typically lower than in IAS
**Insulin-to-C-peptide molar ratio**	Often >1	Typically <1
**IAAs**	Present (diagnostic hallmark)	Absent (rare cases with positive IAAs have been reported)
**Insulin recovery after PEG precipitation**	Low (5–10%)	Normal (>70%)
**Imaging findings**	Imaging studies are generally not required for diagnosis	Pancreatic lesion detectable on abdominal CT, MRI, or 68Ga-DOTATOC PET/CT
**Treatment**	Dietary modification, supportive measures, and/or pharmacologic therapy	Surgical resection of the pancreatic lesion

**Abbreviations:** CT: Computed tomography; IAS: Insulin autoimmune syndrome; IAAs: Insulin autoantibodies; MRI: Magnetic resonance imaging; PEG: Polyethylene glycol; PET/CT: Positron emission tomography/computed tomography.

Beyond insulinoma, several other conditions should be considered in the differential diagnosis of IAS. Although less common, they may warrant consideration in selected clinical scenarios, particularly when the clinical presentation is atypical or diagnostic uncertainty persists despite initial evaluation. Their principal distinguishing features are summarized in Box 1.

Box 1 **Other conditions to consider in the differential diagnosis of IAS**

● **Type B insulin resistance syndrome:** This rare autoimmune disorder is caused by autoantibodies directed against the insulin receptor rather than insulin itself. It typically presents with severe insulin-resistant hyperglycemia and acanthosis nigricans, often in association with systemic autoimmune disease. Of note, isolated hypoglycemia with suppressed insulin has been described as a rare variant. Unlike IAS, IAAs are absent, whereas insulin receptor antibodies are detectable [46].
● **Surreptitious or factitious hypoglycemia:** Administration of exogenous insulin or sulfonylureas should always be considered. Exogenous insulin administration is characterized by elevated insulin concentrations with suppressed C-peptide levels, whereas sulfonylurea-induced hypoglycemia is associated with elevated insulin and C-peptide concentrations together with detectable sulfonylurea metabolites in plasma or urine. IAAs are typically absent in both situations [9,10].
● **Late dumping syndrome:** Reactive hypoglycemia occurring after gastric surgery may mimic the postprandial hypoglycemia pattern observed in IAS. However, the diagnosis is usually evident from the clinical history, and IAAs are not detected [10].

Alt-text: Box 1

### 2.5 Therapeutic management

To date, no standardized treatment guidelines are available for IAS, largely because of its rarity and the absence of randomized controlled trials. Consequently, current management is based primarily on clinical experience, evidence derived from case reports and case series, and expert consensus recommendations. Treatment should therefore be individualized according to the severity of hypoglycemia, symptom burden, and the underlying trigger. When IAS is associated with a specific medication or dietary supplement, prompt withdrawal of the offending agent represents the cornerstone of management [3,4,9]. In a systematic review of 294 patients, 56% achieved remission with dietary modification and trigger removal alone, while 43% needed immunosuppressive therapy [9].

The initial therapeutic approach is primarily supportive and aims to prevent recurrent hypoglycemic episodes while circulating IAA concentrations gradually decline. Nutritional intervention constitutes a key component of treatment and is intended to attenuate postprandial glycemic excursions and reduce the stimulus for excessive insulin secretion. Patients are generally advised to consume small, frequent meals, typically five to six times daily, with particular emphasis on complex carbohydrates and low-glycemic-index foods [3,9,47]. As in other forms of recurrent hypoglycemia, including non-islet cell tumor hypoglycemia, uncooked cornstarch has been used successfully in selected cases. As a slowly absorbed glucose polymer, cornstarch provides a sustained source of glucose and may reduce the risk of delayed hypoglycemic episodes, particularly during fasting periods and overnight [48,49]. However, evidence for cornstarch use in IAS is limited to a single case report, and spontaneous remission could not be excluded [10,50].

When nutritional measures alone fail to provide adequate glycemic control, pharmacological treatment should be considered. One of the most frequently used agents is acarbose, an α-glucosidase inhibitor that delays intestinal carbohydrate absorption, thereby attenuating postprandial hyperglycemia and reducing the subsequent stimulus for excessive insulin secretion. The maximum recommended dose is 100 mg three times daily; however, in individuals weighing less than 60 kg, the dose should not exceed 50 mg three times daily. Gastrointestinal adverse effects, most commonly abdominal discomfort, diarrhea, and flatulence, are frequent, with flatulence reported in up to 74% of cases. These adverse effects are typically dose-dependent, and both their frequency and severity tend to decrease over time [51,52].

Additional pharmacological strategies aimed at suppressing endogenous insulin secretion have also been reported. Diazoxide inhibits insulin secretion through activation of ATP-sensitive potassium (K_ATP) channels via binding to the sulfonylurea receptor 1 (SUR1) subunit, promoting β-cell hyperpolarization and thereby suppressing calcium-dependent insulin exocytosis. However, diazoxide has shown variable efficacy in IAS, and its use may be limited by side effects including fluid retention, hypotension, and tachycardia [12,53]. On the other hand, somatostatin analogs, such as octreotide, inhibit insulin secretion through activation of somatostatin receptors on pancreatic β-cells, resulting in reduced intracellular calcium influx and diminished insulin release [54]. In the acute setting, continuous intravenous glucose infusion may be required to maintain euglycemia, whereas glucagon can be considered in selected patients with severe or refractory hypoglycemia [3].

Patients who continue to experience clinically significant hypoglycemia despite these measures may require immunomodulatory therapy. GCs represent the first-line immunosuppressive treatment and are typically administered as prednisone at a dose of 0.5–1 mg/kg/day or an equivalent GC regimen. GCs are believed to reduce autoantibody production and inhibit binding of autoantibodies to endogenous insulin, while simultaneously enhancing hepatic gluconeogenesis, thereby improving glycemic stability. In patients who do not achieve an adequate response to steroids, alternative immunosuppressive agents may be used either as monotherapy or in combination with GCs [3,4,9].

Based on the available evidence, the need for a second immunosuppressive agent appears to be relatively uncommon in clinical practice. In their systematic review, Patel et al. reported that 78% of patients achieved remission with oral GCs alone, whereas only 22% required additional immunosuppressive therapy [9]. Reported agents include azathioprine, mycophenolate mofetil (MMF), cyclophosphamide, cyclosporine, and rituximab [3,4,9,55]. Among these, rituximab, an anti-CD20 monoclonal antibody, was first used for steroid-refractory IAS in 2016, while its rationale is supported by evidence from T1D prevention trials showing selective suppression of IAAs [56,57].

The duration of immunosuppressive treatment, including GC tapering, generally ranges from one to six months, corresponding to the period during which most patients achieve clinical remission. In severe cases refractory to medical therapy, therapeutic plasma exchange may be considered to rapidly reduce circulating IAA levels and provide prompt relief from hypoglycemia [4,58].

**Fig. 1:**
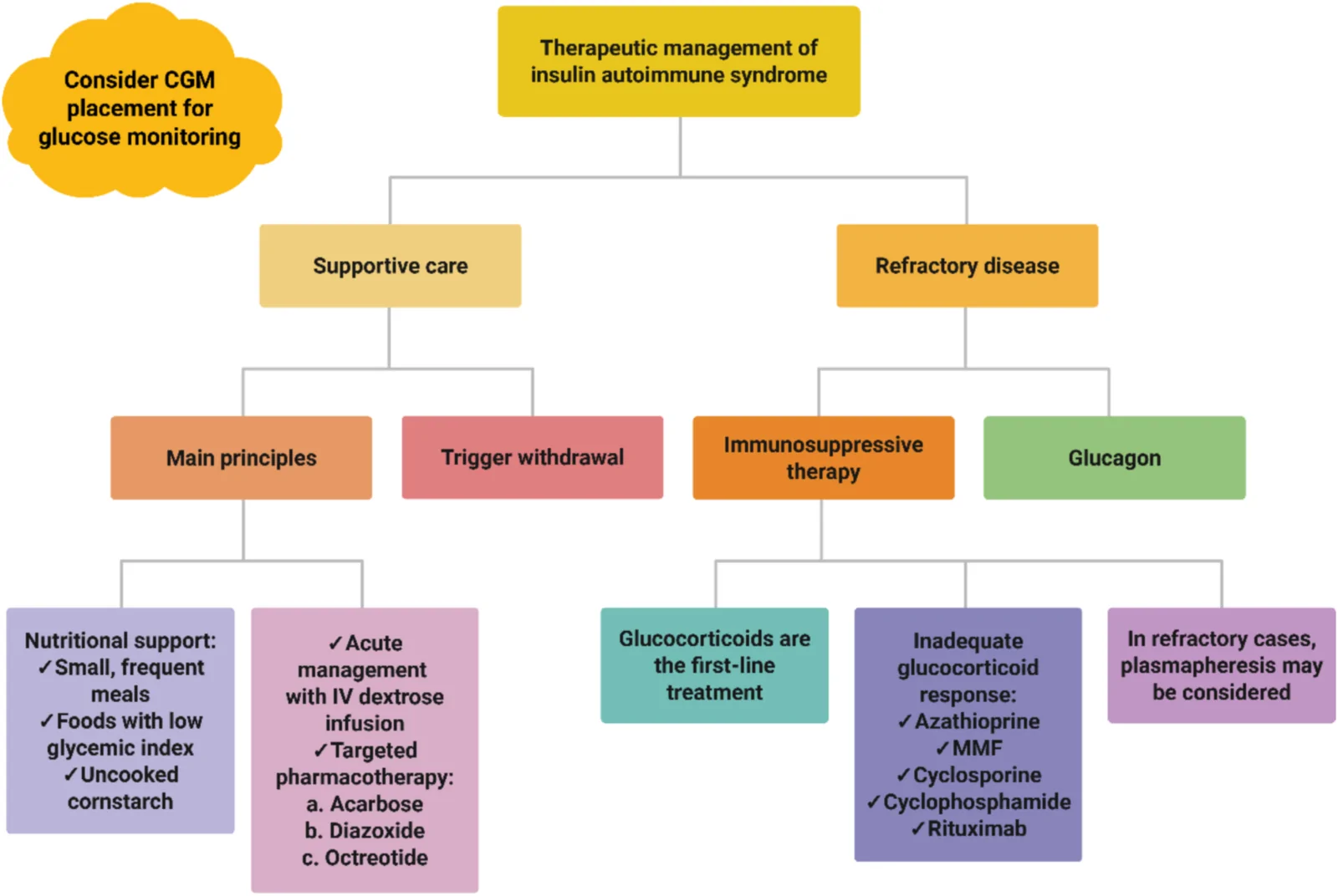
Proposed algorithm for the management of insulin autoimmune syndrome. **Abbreviations:** CGM: Continuous glucose monitoring; IV: Intravenous; MMF: Mycophenolate mofetil. Created in BioRender. Kounatidis, D. (2026) https://BioRender.com/6kecpwy.

Overall, current evidence supports a stepwise therapeutic strategy consisting of withdrawal of the triggering agent, MNT, targeted management of hypoglycemia, and selective use of immunomodulatory therapy in patients with persistent, severe, or refractory disease. Fig. 1 presents a proposed algorithm for the therapeutic management of IAS.

### 2.6 Follow-up and prognosis

CGM may serve as a valuable adjunctive tool both during initial treatment and longitudinal follow-up, enabling real-time detection of hypoglycemic episodes, and facilitating treatment titration [57]. Although no prospective studies have specifically evaluated the role of CGM in IAS, favorable clinical experiences have been reported in individual case reports, suggesting that CGM may support long-term disease monitoring, guide therapeutic decision-making, and contribute to improved clinical management [11,55,59].

The overall prognosis of IAS is generally favorable, with most patients achieving complete remission within weeks to months following withdrawal of the triggering factor and implementation of appropriate supportive measures. The frequently self-limited nature of the syndrome is supported by epidemiological data indicating that approximately 70–80% of patients experience spontaneous resolution within 3–6 months of diagnosis [15,60]. Nevertheless, despite this generally favorable outlook, a subset of patients may experience persistent hypoglycemia, with IAAs remaining detectable in the circulation for several years. The mechanism underlying the dissociation between clinical remission and antibody persistence remains unclear [10]. Finally, recurrence is uncommon but has been reported following re-exposure to the triggering agent or reactivation of an underlying disease such as multiple myeloma [9].

Taken together, follow-up strategies should be individualized according to disease severity, symptom burden, treatment requirements, and the degree of residual glycemic instability.

## 3 Focusing on drug- and supplement-associated IAS

### 3.1 Epidemiology and implicated agents

In a comprehensive analysis integrating pharmacovigilance databases and published case reports, Yu et al. identified 58 medications warranting increased clinical vigilance for the potential development of IAS. Thiol-containing compounds constituted the predominant high-risk category, with methimazole, captopril, and clopidogrel among the most frequently implicated agents. In the same study, proton pump inhibitors (PPIs) and dipeptidyl peptidase-4 (DPP-4) inhibitors also emerged as increasingly recognized causes of drug-induced IAS [23].

Additional evidence has recently been provided by an analysis of 228 IAS cases reported in the FDA Adverse Event Reporting System (FAERS) database between 2004 and 2024. Among the medications evaluated, captopril demonstrated the strongest association with IAS, followed by methimazole and clopidogrel. The study also identified several biological pathways potentially implicated in disease development, including phosphoinositide 3-kinase/protein kinase B (PI3K/Akt) signaling, AMP-activated protein kinase (AMPK) signaling, and insulin resistance–related pathways. Furthermore, a particularly strong association was observed between captopril-associated IAS and the HLA-DRB1*04:06 genotype. Nevertheless, findings derived from FAERS should be interpreted with caution because of the inherent limitations of spontaneous pharmacovigilance databases, including reporting bias, underreporting, incomplete clinical information, and the inability to establish causality [61].

**Fig. 2:**
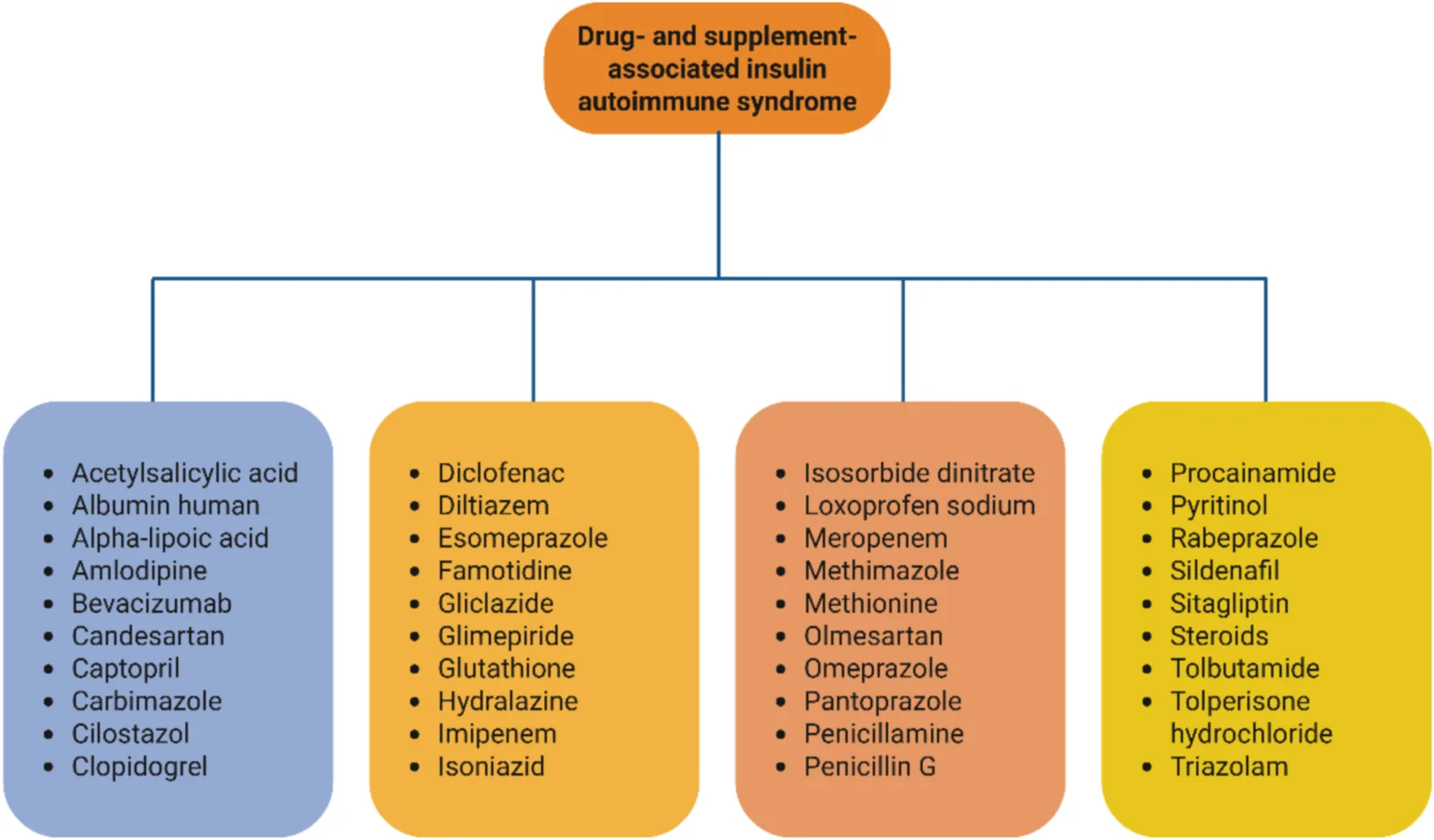
Drug- and supplement-associated insulin autoimmune syndrome. This figure presents drugs and supplements associated with the development of insulin autoimmune syndrome as reported in the literature [3,4,10,23]. Created in BioRender. Kounatidis, D. (2026) https://BioRender.com/qbqbddx.

IAS may also develop in the context of concurrent therapeutic exposures. For example, a recent case report described IAS occurring in a patient receiving concomitant PPI therapy and oxaliplatin-based chemotherapy, suggesting that multiple drug exposures may act synergistically in genetically susceptible individuals [62]. Beyond pharmacological agents, IAS has also been associated with dietary supplements, among which ALA has emerged as one of the most frequently reported and increasingly recognized triggers [3,4,10]. Fig. 2 summarizes medications and supplements reported to be associated with IAS in the published literature.

### 3.2 Pathogenesis

According to the prevailing pathogenic hypothesis, sulfhydryl-containing compounds may reduce the disulfide bonds of the insulin molecule (Cys A6–A11 and Cys B7–B19), inducing conformational changes that expose immunogenic peptide fragments and increase insulin antigenicity. The resulting insulin-derived peptides can subsequently be presented by Major Histocompatibility Complex (MHC) class II molecules, particularly in genetically susceptible individuals carrying specific HLA alleles such as HLA-DRB1*04:06. This process promotes the activation of insulin-reactive CD4^+^ T cells and subsequently stimulates B cells to produce IAAs [10].

Following their formation, IAAs bind a substantial proportion of endogenously secreted insulin after meals, transiently reducing the circulating fraction of free biologically active insulin. Although total serum insulin concentrations become markedly elevated, the amount of insulin available to interact with peripheral insulin receptors is initially insufficient, resulting in early postprandial hyperglycemia. Persistent hyperglycemia subsequently stimulates compensatory insulin secretion from pancreatic β-cells, leading to the formation of large circulating pools of insulin–autoantibody complexes [3,4].

Because the binding of insulin to IAAs is reversible and characterized by low affinity and high binding capacity, these complexes act as a temporary reservoir that sequesters insulin and delays its biological action. Subsequent dissociation of the complexes releases excessive amounts of free insulin into the circulation independently of contemporaneous glucose concentrations, producing inappropriate hyperinsulinemia and ultimately delayed postprandial hypoglycemia. In addition, some patients present predominantly with fasting or early morning hypoglycemia, which may result from overnight dissociation of insulin–autoantibody complexes as antibody binding sites become saturated during the day [10].

Clinically, this mechanism may manifest as marked glycemic variability characterized by alternating episodes of postprandial hyperglycemia and hypoglycemia [3,4,10]. This pathogenic model is supported by a case reported by Dozio et al. in which administration of 125I-labeled insulin demonstrated prolonged intravascular retention of antibody-bound insulin together with absent hepatic and renal uptake, providing direct evidence for the delayed release and altered kinetics of insulin in IAS [63].

## 4 ALA–associated IAS

### 4.1 Biological properties and clinical applications of ALA

ALA is a naturally occurring disulfide compound synthesized within mitochondria that plays an essential role in cellular energy metabolism and redox homeostasis. Together with its reduced form, dihydrolipoic acid (DHLA), it constitutes a potent endogenous antioxidant system capable of scavenging reactive oxygen species (ROS), chelating metal ions, and facilitating the regeneration of other intracellular antioxidants. Owing to these biological properties, ALA has attracted considerable scientific and clinical interest and has been used across a wide range of clinical settings. In contemporary practice, ALA is widely available as an over-the-counter antioxidant supplement, either alone or in combination with vitamins and other nutraceutical compounds [64,65].

Among its clinical indications, ALA is most commonly used in patients with DPN, although its routine use is not universally endorsed by major international guidelines. More recently, increasing interest has focused on its potential role in the prevention and treatment of metabolic dysfunction-associated steatotic liver disease (MASLD) [66,67]. Typical daily doses range from 200 to 600 mg, depending on the indication and individual patient characteristics, although substantially higher doses, reaching up to 1800 mg/day, have also been used in clinical trials [64**–**67].

### 4.2 Current evidence on the association between ALA and IAS

To date, the most comprehensive evaluation of the association between ALA and IAS has been conducted by the European Food Safety Authority (EFSA) Panel on Nutrition, Novel Foods and Food Allergens (NDA). This assessment identified 49 documented cases of ALA-associated IAS reported up to 2021 and remains the most extensive analysis available on this topic [68]. The following subsections summarize and critically appraise the principal findings of this assessment.

#### 4.2.1 Epidemiological characteristics

The age of affected individuals ranged from 28 to 82 years, indicating that ALA-associated IAS may occur across a broad age spectrum. One of the most notable findings of the EFSA assessment was the marked predominance of female patients among reported cases of ALA-associated IAS. This observation is consistent with reports describing a female predominance among patients who developed IAS during methimazole treatment [69]. However, this finding contrasts with the male predominance reported by Yu et al. in their analysis of drug-induced IAS. The authors suggested that this discrepancy may largely reflect regional prescribing patterns, as clopidogrel, one of the medications most strongly associated with IAS, is prescribed predominantly to older men for cardiovascular prevention [23]. The female predominance in ALA-associated IAS may also partly reflect the wider use of ALA-containing supplements among women, particularly in the context of anti-aging and cosmetic supplementation [9,70]. Thus, clinicians should not overlook seeking for various supplementations, especially among females with unexplained hypoglycemia. In this context, the importance of a thoroughly obtained medical history should be pointed out.

Furthermore, the apparent female predominance observed in ALA-associated IAS differs from the broader epidemiological evidence suggesting no clear sex predilection in IAS overall [3,4]. Similar demographic characteristics regarding age at disease onset and sex distribution were reported by Li et al. in another review of published ALA-associated IAS cases. However, interpretation of these findings should take into account the substantial overlap between the two datasets, as many of the patients included in that review had already been incorporated into the EFSA analysis [70].

#### 4.2.2 Genetic susceptibility

Genetic susceptibility appears to play a central role in the development of ALA-associated IAS. In the EFSA analysis, which included both East Asian and Caucasian subjects, a substantial proportion of affected individuals carried specific HLA class II alleles. Among patients who underwent HLA genotyping, HLA-DRB1*04:06 was the most frequently identified allele (19 cases), followed by HLA-DRB1*04:03 (14 cases), whereas HLA-DRB1*04:15 was detected less commonly [68]. In contrast, studies focusing specifically on European and North American populations suggest that HLA-DRB1*04:03 may represent the predominant susceptibility allele, accounting for approximately 67% of reported cases of ALA-associated IAS [71]. These observations are further supported by a small case series of six Caucasian patients with ALA-associated IAS, in which HLA analysis identified HLA-DRB1*04:03 in five patients, whereas HLA-DRB1*04:06 was detected in only one patient [7].

#### 4.2.3 Latency to disease onset and dose-response relationship

The EFSA analysis demonstrated that the interval between initiation of ALA supplementation and the onset of IAS typically ranges from one to four weeks. Nevertheless, delayed presentations occurring up to four months after treatment initiation have also been reported. Importantly, the lowest documented ALA dose associated with IAS development was 200 mg/day. Despite these observations, no clear dose–response relationship was identified between ALA dose and either the risk of disease development or the latency to symptom onset. Similarly, the currently available evidence does not support the existence of a threshold dose below which IAS can be considered unlikely to occur. These findings suggest that individual genetic susceptibility may be a more important determinant of disease development than the administered dose itself [68].

#### 4.2.4 Clinical presentation and therapeutic management

The clinical manifestations and laboratory characteristics of ALA-associated IAS do not appear to differ substantially from those observed in IAS triggered by other medications or supplements. Patients typically present with endogenous hyperinsulinemic hypoglycemia, often accompanied by markedly elevated insulin concentrations and positive IAAs. In the review by Li et al. insulin concentrations were ≥100 μIU/mL in 94.6% of patients, and autonomic symptoms (81.1%) and neuroglycopenic symptoms (64.9%) were the predominant clinical manifestations [70]. Nevertheless, the available literature provides additional insights into the therapeutic management of ALA-associated IAS. Among the 49 patients included in the EFSA analysis, treatment data were available for 28 individuals. In virtually all cases, withdrawal of ALA supplementation and implementation of dietary measures constituted the cornerstone of management [68].

By contrast, pharmacological therapies specifically targeting hypoglycemia were used infrequently [68]. Diazoxide was administered in only two patients, including one who also received prednisone, whereas other agents commonly employed in IAS, such as acarbose and octreotide, were not reported [6,72]. Immunomodulatory treatment was used more frequently, with 16 of the 28 patients receiving GCs. Prednisone was the most commonly prescribed agent, while hydrocortisone was administered in only two cases [68]. Overall, the prognosis of ALA-associated IAS appeared favorable. In most reported cases, hypoglycemia resolved within weeks to months following discontinuation of ALA, with or without additional supportive therapy [68]. IAA titers declined more slowly, with a median time to seronegativity of 7 months (range 2–36 months), consistent with the long half-life of Ig antibodies [68].

#### 4.2.5 Clinical implications

Based on the available evidence, the EFSA Panel concluded that consumption of ALA-containing dietary supplements and foods is likely to increase the risk of IAS in genetically susceptible individuals. However, because susceptibility-associated HLA polymorphisms cannot be readily identified in routine clinical practice and because the available epidemiological data remain limited, the absolute risk associated with ALA exposure cannot currently be quantified with precision [68]. From a clinical perspective, these findings highlight the importance of obtaining a detailed history of dietary supplement use in patients presenting with unexplained hyperinsulinemic hypoglycemia. Particular attention should be paid to ALA exposure, especially in populations known to carry HLA alleles associated with increased susceptibility to IAS.

### 4.3 Published cases of ALA-associated IAS reported between 2022 and 2026

A literature review of the PubMed, Embase, and Scopus databases identified twelve cases of ALA-associated IAS during the last five years, published between January 2022 and 24 May 2026. Table 2 summarizes the demographic characteristics, biochemical findings, therapeutic interventions, and clinical outcomes of these patients.

**Table 2:** Reported cases of alpha-lipoic acid–associated insulin autoimmune syndrome published during the last 5 years between 2022 and 2026.

Author, year	Case (age, sex, and comorbidities)	ALA dose and symptom-onset interval	Symptoms/Diagnosis	Treatment/outcome
Sehgal, 2023 [73]	✔ A 51-year-old non-diabetic male ✔ No comorbidities	✔ Dose NS ✔ 1 month	✔ Repeated episodes of sweating, anxiety, palpitations, and lethargy, both during the fasting and postprandial state, for 20 days ✔ Serum glucose: 44 mg/dL ✔ C-peptide: 12.8 ng/ml ✔ Serum insulin: 1000 μIU/mL ✔ IAAs: >100 U/mL	✔ ALA discontinuation ✔ IV dextrose infusion ✔ MNT and prednisolone 60 mg/daily with tapering over 2 months ✔ Clinical remission
Βaburaj, 2024 [74]	✔ A 40-year-old non-diabetic female ✔ No comorbidities	NS	✔ Coma ✔ Serum glucose: very low (NS) ✔ C-peptide: elevated (NS) ✔ Serum insulin: high (NS) ✔ IAAs: positive (NS)	✔ IV dextrose infusion ✔ Steroids (NS) ✔ Clinical remission
Uysal, 2024 [75]	✔ A 60-year-old non-diabetic female ✔ History of migraine for 30 years	✔ 600 mg daily ✔ About 1 month	✔ Hunger, dizziness, sweating, tremor, and palpitations for 1 month ✔ Serum glucose: 31 mg/dL ✔ C-peptide: 28.2 ng/ml ✔ Serum insulin >1000 μIU/mL ✔ IAAs >175 IU/mL	✔ ALA discontinuation ✔ Methylprednisolone 32 mg/day with gradual tapering to 4 mg/day within 2 months ✔ Clinical remission
Mudagall, 2025 [76]	✔ A 48-year-old male ✔ Comorbidities NS	NS	✔ Recurrent episodes of hypoglycemia (both fasting and postprandial) for 2 months. Main symptoms included palpitation, tremors, as well as one episode of altered sensorium requiring hospitalization ✔ Serum glucose: 40 mg/dL ✔ C-peptide: 32.3 ng/ml ✔ Serum insulin >1000 μIU/mL ✔ IAAs: 85.3 IU/mL	✔ ALA discontinuation ✔ MNT with acarbose 25 mg 3 times/daily with meals for 3 months ✔ Clinical remission
Mudagall, 2025 [76]	✔ A 38-year-old female ✔ Comorbidities NS	NS	✔ Recurrent postprandial hypoglycemia (symptoms NS) for 1 month ✔ Serum glucose: 29 mg/dL ✔ C-peptide: 23.96 ng/ml ✔ Serum insulin >1000 μIU/mL ✔ ΙΑΑs: 188 IU/mL	✔ ALA discontinuation ✔ IV dextrose infusion ✔ MNT ✔ Prednisolone 40 mg/day, tapered for a month ✔ Clinical remission
Mudagall, 2025 [76]	✔ A 42-year-old female ✔ Comorbidities NS	NS	✔ Multiple episodes of postprandial hypoglycemia (symptoms NS) for 5 months ✔ Glucose: 20 mg/dL during OGTT ✔ C-peptide: 5.5 ng/ml ✔ Serum insulin: 600 μIU/mL ✔ IAAs: 78.7 IU/mL	✔ ALA discontinuation ✔ MNT with acarbose 25 mg 3 times daily with meals and ✔ Prednisolone 40 mg/day ✔ Clinical outcome not reported
Mudagall, 2025 [76]	✔ A 60-year-old female ✔ History of nephrolithiasis	NS	✔ Multiple episodes of postprandial hypoglycemia (symptoms NS) for 20 days ✔ Serum glucose: 20 mg during OGTT ✔ C-peptide: 23.02 ng/ml ✔ Serum insulin >1000 μIU/mL ✔ IAAs: 98 IU/mL	✔ ALA discontinuation ✔ MNT with acarbose 25 mg 3 times/daily with meals for 6 months ✔ Clinical remission
Mudagall, 2025 [76]	✔ A 64-year-old male ✔ History of hypertension under treatment with telmisartan, amlodipine, and chlorthalidone	✔ Dose NS ✔ 2 months	✔ Episodes of recurrent postprandial hypoglycemia (symptoms not NS) for 1 month ✔ Serum glucose: 34 mg/dL ✔ C-peptide: 23.27 ng/ml ✔ Serum insulin >1000 μIU/mL ✔ IAAs: 93 IU/mL	✔ ALA discontinuation ✔ MNT with acarbose 25 mg 3 times/daily with meals for 3 months ✔ Clinical remission
Viti, 2025 [77]	✔ A 25-year-old ✔ female in the 24th week of her first pregnancy ✔ History of celiac disease and Hashimoto thyroiditis	✔ 600 mg twice daily ✔ 2 weeks	✔ Recurrent hypoglycemia, characterized by tremors, sweating, and palpitations, that began at 20 weeks of pregnancy, followed by an episode of severe hypoglycemia, which resulted in syncope ✔ Serum glucose: 18 mg/dL ✔ C-peptide: 6.3 ng/mL ✔ Serum insulin: 87 μIU/mL ✔ IAAs >100 IU/mL	✔ ALA discontinuation ✔ MNT and prednisolone 25 mg/daily with tapering between 27th week and 30th week of pregnancy ✔ Postpartum outcome: a. Maternal clinical remission b. Neonatal hypoglycemia (<47 mg/dL) in the first weeks of life, with IAA levels of 61 U/mL on the fifth day of life. Gradual remission of episodes.
Subramaniam, 2025 [78]	✔ A 57-year-old female ✔ No comorbidities	✔ 300 mg twice daily ✔ 3 weeks, with a 2-week discontinuation interval	✔ Nocturnal postprandial hypoglycemic episodes with palpitations and sweating; disorientation prompted ED admission ✔ Serum glucose: 44 mg/dL ✔ C-peptide: 6.2 ng/mL ✔ Serum insulin >200 μU/mL ✔ IAAs: 345 mU/L	✔ ALA discontinuation ✔ IV dextrose infusion ✔ MNT and prednisolone 30 mg/daily with tapering over 5 months ✔ Clinical remission
Subramaniam, 2025 [78]	✔ A 55-year-old male ✔ History of obesity	✔ 300 mg daily ✔ 12 days, with a 1-week discontinuation interval	✔ Episodes of anxiety, hunger, and palpitations, predominantly during the early morning hours ✔ Serum glucose: 47 mg/dL ✔ C-peptide: 5.1 ng/mL ✔ Serum insulin: 2104 μU/mL ✔ IAAs: 234 mU/L	✔ ALA discontinuation ✔ MNT ✔ Clinical remission after 1 month
AbuBaha, 2026 [79]	✔ A 35-year-old female ✔ No comorbidities	✔ Dose NS ✔ 13 days	✔ Dizziness, profuse sweating, tachycardia, shortness of breath, and a nocturnal seizure episode (following acute urticarial allergic reaction treated with bilastine) ✔ Fasting serum glucose: 38–41 mg/dL ✔ Postprandial serum glucose: 34–58 mg/dL ✔ C-peptide: 5.04 ng/mL ✔ Serum insulin >2778 μU/mL ✔ IAAs: 43.0 U/ml	✔ MNT ✔ 6-month immunosuppressive regimen: prednisolone 35–40 mg/day, gradually tapered after azathioprine 50 mg/day was added 10 days later ✔ Clinical remission

**Abbreviations:** ALA: Alpha-lipoic acid; IAAs: Insulin autoantibodies; ED: Emergency department; IV: Intravenous; MNT: Medical nutrition therapy; NS: Not specified; OGTT: Oral Glucose Tolerance Test.

Although the number of reported cases remains insufficient to permit robust comparisons with the EFSA analysis, several clinically relevant observations are noteworthy. First, while only a minority of reports provided detailed information regarding the interval between ALA initiation and symptom onset, the available data suggest a variable latency period. In some patients, hypoglycemia developed after 1–2 months of ALA exposure, consistent with the EFSA findings [73,75,76]. In contrast, other reports documented symptom onset within less than two weeks of treatment initiation [[77], [78], [79]]. Interestingly, in two cases, hypoglycemic symptoms developed despite temporary discontinuation of ALA shortly after treatment initiation, suggesting that disease onset may emerge even after cessation of exposure in susceptible individuals [78]. Among cases reporting dosage information, daily ALA doses ranged from 300 to 1200 mg [75,77,78].

The clinical presentation was broadly consistent with that described in previous reports of IAS. Most patients presented with autonomic manifestations of hypoglycemia, whereas neuroglycopenic symptoms were reported less frequently [[73], [74], [75]]. Hypoglycemic episodes were predominantly postprandial [73,76], although fasting hypoglycemia was also documented in selected cases [76,79]. Severe hypoglycemia was common, with one patient presenting with a plasma glucose concentration of 18 mg/dL, representing the lowest reported value in this series [77]. Marked variability in serum insulin concentrations was observed; however, insulin levels exceeded 1000 μIU/mL in several patients, consistent with the substantial hyperinsulinemia frequently observed in IAS [75,76,78,79].

With regard to treatment, acarbose was used successfully in several patients, particularly in the case series reported by Mudagall et al. without treatment discontinuation due to adverse effects [76]. GC therapy was also administered in selected cases with favorable clinical responses [75]. On the other hand, advanced immunosuppressive therapy was rarely required, with only one patient receiving azathioprine as a steroid-sparing agent [79]. This is consistent with both the EFSA analysis and previous reports indicating that second-line immunosuppressive therapy is seldom required in ALA-associated IAS [9,68].

An additional clinically important observation emerged from the case of ALA-associated IAS occurring during pregnancy, highlighting the need for vigilance regarding neonatal hypoglycemia in pregnancies complicated by IAS. Specifically, persistent neonatal hypoglycemia was observed despite resolution of maternal glycemic abnormalities after delivery, a phenomenon attributed to transplacental transfer of maternal IAAs. Close glucose monitoring and appropriate nutritional support were sufficient to achieve complete recovery [77].

Collectively, these cases reinforce the current understanding that ALA-associated IAS generally follows a favorable clinical course. Most patients achieve remission following discontinuation of ALA, implementation of individualized nutritional strategies, and selective use of pharmacological therapy when required. Notably, with the exception of a single patient treated with azathioprine, neither advanced immunosuppressive therapy nor therapeutic plasma exchange was required.

## 5 Translating evidence into clinical practice: A case of ALA–Associated IAS

A 76-year-old man was admitted to our hospital because of altered mental status accompanied by lethargy, which developed during the early morning hours shortly after awakening. His medical history was notable for dyslipidemia, Parkinson's disease, chronic obstructive pulmonary disease, and hypothyroidism. His regular medications included rosuvastatin 20 mg daily, levodopa/benserazide hydrochloride 200/50 mg, inhaled salbutamol/ipratropium twice daily, and levothyroxine 62 μg daily. Upon presentation to the Emergency Department (ED), the patient was lethargic but afebrile. Arterial blood gas analysis revealed no significant abnormalities other than hypoglycemia, with a plasma glucose concentration of 42 mg/dL. Intravenous administration of 10% dextrose resulted in prompt restoration of normal mental status, thereby fulfilling Whipple's triad.

Given the absence of an apparent explanation for the hypoglycemic episode, serum insulin and C-peptide concentrations were measured. C-peptide was elevated at 10.11 ng/mL (reference range: 0.8–4.2 ng/mL), while serum insulin was markedly increased at 1126 μIU/mL (reference range: 2.6–24.9 μIU/mL), confirming endogenous hyperinsulinemia. In the absence of exposure to glucose-lowering medications, the differential diagnosis primarily included insulinoma and IAS. Further diagnostic evaluation consisted of serum IAA measurement and pancreatic imaging, which was performed because of the prolonged turnaround time required for IAA testing. The PET/CT examination showed no evidence of a pancreatic neuroendocrine tumor, whereas serum IAAs were strongly positive, with a titer >175 IU/mL (reference range: <20 IU/mL). Based on these findings, a diagnosis of IAS was established.

No obvious triggering factor was initially identified. However, careful reassessment of the patient's medication and supplement history revealed that he had started taking ALA 600 mg daily as a dietary supplement approximately one month before symptom onset. This latency is consistent with the typical interval of one to four weeks reported in the EFSA analysis [68]. The supplement had already been discontinued upon hospital admission. In addition to intravenous glucose administration, the patient received individualized medical nutrition therapy (MNT) consisting of frequent, small, low-glycemic-index meals. Despite these measures, he continued to experience recurrent hypoglycemic episodes, including both fasting and postprandial hypoglycemia. Consequently, treatment with methylprednisolone 32 mg daily was initiated. This dose is equivalent to approximately 40 mg of prednisone, consistent with the commonly recommended range of 0.5–1 mg/kg/day [10]. GC treatment was gradually tapered over a six-month period. With this approach, the patient remained asymptomatic, and no further episodes of hypoglycemia were detected on continuous CGM, which was initiated at hospital discharge. The use of CGM in this case is supported by emerging evidence suggesting its utility in quantifying glycemic excursions and evaluating treatment efficacy in IAS [57].

This case illustrates several key principles in the diagnosis and management of IAS. It highlights the importance of meticulous reassessment of medication and dietary supplement exposure, particularly when no apparent trigger is identified during the initial evaluation. Furthermore, it demonstrates how integration of current evidence-based strategies can facilitate disease remission and lead to favorable clinical outcomes. Notably, the insulin concentration of 1126 μIU/mL observed in this patient is consistent with the markedly elevated levels typically reported in IAS, with ≥100 μIU/mL documented in 94.6% of ALA-associated cases in the Li et al. review [70]. The patient's clinical presentation, biochemical findings, treatment, and outcome are summarized in Table 3.

**Table 3:** Clinical, biochemical, and therapeutic characteristics of the patient.

Characteristic	Finding
**Sex**	Male
**Age**	76 years
**Triggering agent**	Alpha-lipoic acid, 600 mg daily, initiated 1 month before presentation
**HLA genotyping**	Not performed (not available)
**Serum glucose at presentation**	42 mg/dL
**C-peptide during hypoglycemia**	10.11 ng/mL (reference range: 0.8–4.2 ng/mL)
**Proinsulin during hypoglycemia**	Not measured (not available)
**Serum insulin concentration**	1126 μIU/mL (reference range: 2.6–24.9 μIU/mL)
**Insulin-to-C-peptide molar ratio**	2.02
**Insulin autoantibodies (IAAs)**	>175 IU/mL (reference range: <20 IU/mL)
**Pancreatic imaging**	68Ga-DOTATOC PET/CT: no evidence of pancreatic neuroendocrine tumor
**Treatment**	Discontinuation of alpha-lipoic acid; medical nutrition therapy; methylprednisolone 32 mg daily with gradual tapering over 6 months
**Follow-up**	Continuous glucose monitoring (CGM) for 6 months
**Outcome**	Complete clinical remission

## 6 Conclusions

IAS is a rare but increasingly recognized cause of endogenous hyperinsulinemic hypoglycemia. Although initially described predominantly in East Asian populations, the growing number of cases reported worldwide suggests that the condition is likely underdiagnosed rather than exceptionally uncommon. Accurate diagnosis relies on recognition of the characteristic clinical and biochemical features of the syndrome, careful assessment of medication and supplement exposure, and appropriate measurement of IAAs. Current evidence supports a stepwise management approach based on withdrawal of the triggering factor, individualized MNT, targeted treatment of hypoglycemia, and selective use of immunomodulatory therapy in patients with persistent or severe disease.

Among the various precipitating factors, ALA has emerged as one of the most clinically relevant triggers, particularly in non-East Asian populations, reflecting the widespread and often unsupervised use of this dietary supplement. The case presented herein demonstrates how timely recognition of ALA-associated IAS and application of evidence-based management principles can prevent unnecessary investigations, such as unwarranted surgical exploration for suspected insulinoma, guide appropriate therapeutic decisions, and result in favorable clinical outcomes. As the use of ALA and other potential triggers continues to expand, increased clinician awareness and a high index of suspicion in patients presenting with unexplained hyperinsulinemic hypoglycemia will be essential for ensuring prompt diagnosis and optimal patient care.

## CRediT authorship contribution statement

**Natalia G. Vallianou:** Conceptualization, Data curation, Resources. **Dimitris C. Kounatidis:** Formal analysis, Methodology, Project administration. **Maria Dalamaga:** Formal analysis, Investigation, Writing – review & editing. **Fotis Panagopoulos:** Data curation, Resources, Validation. **Maria Psaroudaki:** Data curation, Formal analysis, Investigation. **Ioanna Tantsi:** Data curation, Resources, Validation. **Theodora Stratigou:** Data curation, Investigation, Methodology, Resources.

## Informed consent statement

Informed consent for publication of this case was obtained from the patient.

## Institutional review board statement

Not applicable.

## Data availability statement

Not applicable.

## Funding

This research received no external funding.

## Declaration of competing interests

Given her role as co-Editor-in-chief, Prof Maria Dalamaga had no involvement in the peer review of this article and had no access to information regarding its peer review. Full responsibility for the editorial process regarding this article was delegated to another journal editor. The rest of the authors declare that they have no known competing financial interests or personal relationships that could have appeared to influence the work reported in this paper.

## Abbreviations

Akt: Protein kinase B
ALA: Alpha-lipoic acid
AMPK: AMP-activated protein kinase
CGM: Continuous glucose monitoring
CT: Computed tomography
DHLA: Dihydrolipoic acid
DPN: Diabetic peripheral neuropathy
DPP-4: Dipeptidyl peptidase-4
ED: Emergency department
EFSA: European Food Safety Authority
FAERS: FDA Adverse Event Reporting System
GCs: Glucocorticoids
GLP-1: Glucagon-like peptide-1
HLA: Human Leukocyte Antigen
IAAs: Insulin autoantibodies
IAS: Insulin autoimmune syndrome
Ig: Immunoglobulin
K_ATP: ATP-sensitive potassium
MASLD: Metabolic dysfunction-associated steatotic liver disease
MGUS: Monoclonal gammopathy of undetermined significance
MHC: Major Histocompatibility Complex
MMF: Mycophenolate mofetil
MNT: Medical nutrition therapy
MRI: Magnetic resonance imaging
NDA: Nutrition, Novel Foods and Food Allergens
OGTT: Oral Glucose Tolerance Test
PEG: Polyethylene glycol
PET/CT: Positron emission tomography/computed tomography
PI3K: Phosphoinositide 3-kinase
PPIs: Proton pump inhibitors
ROS: Reactive oxygen species
SARS-CoV-2: Severe acute respiratory syndrome coronavirus 2
SSTR: Somatostatin receptor
SUR1: Sulfonylurea receptor 1
T1D: Type 1 diabetes
T2D: Type 2 diabetes
